# The Vagaries of Typhoid

**Published:** 1899-04-08

**Authors:** 


### THE VAGARIES OF TYPHOID.

It is well to keep ourselves always thoroughly awake to the vagaries of the typhoid bacillus, and not to tie ourselves down to abstract definitions of the disease which it sets up, especially in view of the fact that the names by which the malady is officially recognised, both in this country and abroad, seem to imply that typhoid fever is an essentially abdominal or bowel disease. An important paper is published by Dr. Bryant[1] on a case of typhoid fever without any lesion of the intestine, which, nevertheless, gave the Widal reaction during life, and from which the bacillus typhi abdominalis was obtained by culture from the enlarged mesenteric glands found at the necropsy. The patient was a child one year and nine months old, in whom diarrhœa and vomiting had come on two weeks before, and still continued at the time of admission. He died at what was estimated to be about the end of the third week from the onset of the disease. It was anticipated that at the necropsy the usual typical ulceration of Peyer's patches of the lower part of the ileum would be found; but no swelling, discolouration, ulceration, or other abnormalities whatsoever could be detected in the Peyer's patches, solitary glands, or mucous membrane of any part of the intestine. Some enlarged glands were, however, found in the mesentery, and cultures from these yielded an almost pure culture of the bacillus typhi abdominalis. Osler says, in his "Principles and Practice of Medicine": "The wide existence of the typhoid bacillus has been repeatedly shown in cases which had the clinical feature of the disease, but without lesions in the small intestine. Typhoid fever is no more primarily intestinal than is small-pox primarily a cutaneous disease," and he thinks that there may be a condition which he terms "typhoid septicæmia," a general infection with the bacilli without special local manifestations. Other writers also have recorded cases of typhoid fever without intestinal lesions, but the diagnosis of these cases has rested chiefly on clinical evidence without bacteriological verification. Perhaps the most interesting point that arises in regard to the case described by Dr. Bryant is the important vindication which it offers of the great value of a positive Widal reaction, and of the necessity of making a careful bacteriological investigation before excluding the presence of typhoid fever, even when a post mortem examination has been made; for it seems clear that in this case, if cultures had not been made from the enlarged mesenteric glands, the cause of death would in all probability have been assigned to the broncho-pneumonia which manifestly existed, and thus the diagnostic value of the serum reaction as an indication of typhoid fever would have been depreciated.

[1] Brit. Med. Jour., April 1.

